# Uncovering Differences in Virulence Markers Associated with Achromobacter Species of CF and Non-CF Origin

**DOI:** 10.3389/fcimb.2017.00224

**Published:** 2017-05-29

**Authors:** Brankica Filipic, Milka Malesevic, Zorica Vasiljevic, Jovanka Lukic, Katarina Novovic, Milan Kojic, Branko Jovcic

**Affiliations:** ^1^Faculty of Pharmacy, University of BelgradeBelgrade, Serbia; ^2^Institute of Molecular Genetics and Genetic Engineering, University of BelgradeBelgrade, Serbia; ^3^The Institute for Health Protection of Mother and Child SerbiaBelgrade, Serbia; ^4^Faculty of Biology, University of BelgradeBelgrade, Serbia

**Keywords:** cystic fibrosis, *Achromobacter* spp., *nrdA*, antimicrobial resistance, virulence traits

## Abstract

*Achromobacter* spp. are recognized as emerging pathogens in hospitalized as well as in cystic fibrosis (CF) patients. From 2012 to 2015, we collected 69 clinical isolates (41 patient) of *Achromobacter* spp. from 13 patients with CF (CF isolates, *n* = 32) and 28 patients receiving care for other health conditions (non-CF isolates, *n* = 37). Molecular epidemiology and virulence potential of isolates were examined. Antimicrobial susceptibility, motility, ability to form biofilms and binding affinity to mucin, collagen, and fibronectin were tested to assess their virulence traits. The *nrdA* gene sequencing showed that *A. xylosoxidans* was the most prevalent species in both CF and non-CF patients. CF patients were also colonized with *A. dolens/A. ruhlandii, A. insuavis*, and *A. spiritinus* strains while non-CF group was somewhat less heterogenous, although *A. insuavis, A. insolitus*, and *A. piechaudii* strains were detected beside *A. xylosoxidans*. Three strains displayed clonal distribution, one among patients from the CF group and two among non-CF patients. No significant differences in susceptibility to antimicrobials were observed between CF and non-CF patients. About one third of the isolates were classified as strong biofilm producers, and the proportion of CF and non-CF isolates with the ability to form biofilm was almost identical. CF isolates were less motile compared to the non-CF group and no correlation was found between swimming phenotype and biofilm formation. On the other hand, CF isolates exhibited higher affinity to bind mucin, collagen, and fibronectin. In generall, CF isolates from our study exhibited *in vitro* properties that could be of importance for the colonization of CF patients.

## Introduction

*Achromobacter* spp. are recognized as emerging pathogens that can cause infections in patients with impaired immune system and are well-known nosocomial pathogens, especially in the intensive care units (ICUs). Also, *Achromobacter* spp. have been increasingly isolated from respiratory secretions of individuals with cystic fibrosis (CF), in which genetic disorder causes accumulation of thick sticky mucus, mostly in the lungs, leading to frequent bacterial pulmonary infections (Ronne Hansen et al., 2006; Amoureux et al., 2013).

*A. xylosoxidans* is the predominantly isolated *Achromobacter* species from the CF clinical samples. The prevalence of *A. xylosoxidans* in CF patients ranges from 2% to up to 21.8% as reported by a Brazilian CF center (Tan et al., 2002; Pereira et al., 2011); other authors reported an increase in prevalence as well (Emerson et al., 2010; Ridderberg et al., 2011). The frequency of *A. xylosoxidans* isolation in some European centers is relatively high, e.g., 16% in an Italian CF center (Trancassini et al., 2014) and 17.5% in a CF center in France (Amoureux et al., 2013).

Apart from increased prevalence, *A. xylosoxidans* clinical isolates demonstrated broad-spectrum multiple drug resistance, though not uniformly. Intrinsic and acquired resistance tend to limit the choice of antibiotics that can be successfully used in the treatment of infections caused by this bacterium.

Other *Achromobacter* species, namely *A. ruhlandii, A. piechaudii, A. denitrificans, A. spanius, A. insolitus* and *A. marplatensis* have been less frequently associated with CF lung disease comparing to *A. xylosoxidans* (Coenye et al., 2003; Gomila et al., 2011). However, as epidemiology, clinical impact and antibiotic resistance patterns of these novel *Achromobacter* species in CF patients are still unknown, they are the subject of the expanding interest.

Despite their more frequent isolation and ever-emerging multidrug-resistant strains, little is known regarding the virulence characteristics of *Achromobacter*. The mechanisms that make *Achromobacter* species able to adhere, colonize and subsequently infect the respiratory tract are still unclear. Therefore, investigation of the organization and expression of virulence factors is important, particularly as the incidence of *Achromobacter* infections continues to rise.

Over the past few years, *Achromobacter* spp. have been recovered with increasing frequency at the Mother and Child Health Care Institute of Serbia “Dr Vukan Čupić,” a 400-bed university-affiliated pediatric tertiary care hospital in Belgrade, Serbia. The National Cystic Fibrosis Center located within the hospital complex provides multidisciplinary care to outpatients and inpatients of all ages. During the two-and-a-half-year period (from November 2012 to March 2015), we prospectively collected 69 clinical *Achromobacter* spp. isolates from patients with CF (CF isolates) and patients receiving care for other health conditions (non-CF isolates).

The aims of this study were to unveil the molecular epidemiology and the virulence potential of the *Achromobacter* spp. of CF and non-CF origin in a large Serbian clinical setting.

## Materials and methods

### Patients

During the study period (November 2012 to March 2015), *Achromobacter* spp. were recovered from clinical samples obtained from 41 patients (18 males and 23 females, male:female ratio 1:1.25). Among them, 13 were CF patients treated as outpatients and inpatients (the average age was 7.5 years, range 0–30 years). Out of 28 patients treated for health disorders not related to CF (non-CF group, average age 4.4 years, range 0–17 years), 20 (71.4%) were hospitalized in two ICUs (pediatric medical and surgical ICU), while the remaining 8 were treated on different specialized clinical wards.

### Bacterial strains

69 *Achromobacter* spp. clinical isolates were collected. Thirty-two CF isolates were cultured from sputum (*n* = 19), cough swabs (*n* = 7), bronchoalveolar lavage fluid (BALF) (*n* = 3) and blood (*n* = 3) (Table 1). In total, 24 isolates were recovered from chronically colonized patients (6 patients) and 8 isolates were recovered from transient colonization (7 patients) (Table 1). Chronic colonization was defined as at least three positive cultures per year, with a minimum 1-month interval between them, for at least 2 years (Pereira et al., 2011). Thirty-seven non-CF isolates were cultured from a number of sites, including tracheal aspirate (*n* = 25), BALF (*n* = 4), sputum (*n* = 4), blood (*n* = 2), nasal swab (*n* = 1), and ear swab (*n* = 1) (Table 2). These strains were collected from colonized non-CF patients, and in majority of them the prolonged carriage was noted.

**Table 1:** **Labeling, date of sampling, origin and strain type of CF isolates**.

**No of patient**	**Date of sampling (month/year)**	**Isolate**	**Specimen/Colonization status**	**Species**	**No of patient**	**Date of sampling(month/year)**	**Isolate**	**Specimen/Colonization status**	**Species**	**No of patient**	**Date of sampling(month/year)**	**Isolate**	**Specimen Colonization status**	**Species**
1	11/2012	MS4	Sputum/chronic	*A. dolens*/*A. ruhlandii*	4	10/2013	10448	Cough swab/transient	*A. spiritinus*	10	10/2014	12281	Sputum/transient	*A. xylosoxidans*
1	11/2012	MS9	Sputum/chronic	*A. dolens*/*A. ruhlandii*	4	02/2014	1874	Cough swab/transient	*A. insuavis*	11	12/2014	13949	Sputum/chronic	*A. xylosoxidans*
1	06/2013	5657B	Sputum/chronic	*A. dolens*/*A. ruhlandii*	5	01/2014	1074	Cough swab/transient	*A. xylosoxidans*	11	01/2015	1115	Sputum/chronic	*A. xylosoxidans*
1	06/2013	5657C	Sputum/chronic	*A. dolens*/*A. ruhlandii*	6	11/2014	10412	Cough swab/chronic	*A. xylosoxidans*	11	01/2015	1115/2	Sputum/chronic	*A. xylosoxidans*
2	12/2012	FB1	Sputum/chronic	*A. xylosoxidans*	6	02/2015	1701	Cough swab/chronic	*A. xylosoxidans*	12	01/2015	3F	BALF/chronic	*A. xylosoxidans*
2	12/2012	FB2	Sputum/chronic	*A. xylosoxidans*	7	09/2014	1000A	Sputum/chronic	*A. xylosoxidans*	12	01/2015	282	Blood/chronic	*A. xylosoxidans*
2	07/2013	7224A	Sputum/chronic	*A. xylosoxidans*	7	09/2014	1000B	Sputum/chronic	*A. xylosoxidans*	12	02/2015	37F	BALF/chronic	*A. xylosoxidans*
2	07/2013	7224C	Sputum/chronic	*A. xylosoxidans*	7	12/2014	13227	Sputum/chronic	*A. xylosoxidans*	12	02/2015	774	Blood/chronic	*A. xylosoxidans*
2	02/2015	1411A	sputum/chronic	*A. xylosoxidans*	7	02/2015	2244	Sputum/chronic	*A. xylosoxidans*	12	03/2015	1113	Blood/chronic	*A. xylosoxidans*
2	02/2015	1411C	Sputum/chronic	*A. xylosoxidans*	8	10/2014	11304	Cough swab/transient	*A. xylosoxidans*	13	10/2014	10118	Cough swab/transient	*A. xylosoxidans*
3	07/2013	7955	Sputum/transient	*A. xylosoxidans*	9	10/2014	298FA	BALF^1^/transient	*A. xylosoxidans*					

1 BALF, bronchoalveolar lavage fluid

**Table 2:** **Labeling, date of sampling, origin, and strain type of non-CF isolates**.

**No of patient**	**Date of sampling (month/year)**	**Isolate**	**Specimen**	**Strain**	**No of patient**	**Date of sampling (month/year)**	**Isolate**	**Specimen**	**Strain**	**No of patient**	**Date of sampling (month/year)**	**Isolate**	**Specimen**	**Strain**
14	01/2013	A1	TA1	*A. xylosoxidans*	24	11/2013	12330	TA	*A. xylosoxidans*	35	10/2014	10819	ear swab	*A. xylosoxidans*
15	01/2013	A2	TA	*A. xylosoxidans*	25	10/2013	320 FA	BALF	*A. xylosoxidans*	36	10/2014	296FA	BALF	*A. xylosoxidans*
16	03/2013	A3	BALF2	*A. xylosoxidans*	25	11/2013	12051	TA	*A. xylosoxidans*	37	11/2014	11522	TA	*A. xylosoxidans*
16	05/2013	5226	TA	*A. xylosoxidans*	25	12/2013	12969	TA	*A. xylosoxidans*	37	12/2014	13463	TA	*A. xylosoxidans*
17	04/2013	A4	BALF	*A. xylosoxidans*	26	10/2013	11462	TA	*A. xylosoxidans*	38	01/2015	68	TA	*A. xylosoxidans*
18	04/2013	A5	Nasal swab	*A. insolitus*	27	01/2014	651/14	TA	*A. xylosoxidans*	39	01/2015	507	TA	*A. xylosoxidans*
19	04/2013	A6	Sputum	*A. xylosoxidans*	28	02/2014	1841	TA	*A. xylosoxidans*	40	01/2015	913	TA	*A. xylosoxidans*
19	05/2013	MC10	Sputum	*A. xylosoxidans*	29	05/2014	5148	TA	*A. xylosoxidans*	40	02/2015	2367	TA	*A. xylosoxidans*
20	06/2013	6651	Sputum	*A. insuavis*	30	05/2014	5158	TA	*A. xylosoxidans*	41	01/2015	910/2	TA	*A. xylosoxidans*
21	07/2013	7491	Sputum	*A. piechaudii*	31	06/2014	2412	blood	*A. xylosoxidans*	41	01/2015	910	TA	*A. xylosoxidans*
22	07/2013	7498	TA	*A. xylosoxidans*	32	09/2014	3565	blood	*A. xylosoxidans*	41	03/2015	2400	TA	*A. xylosoxidans*
23	08/2013	8361	TA	*A. xylosoxidans*	33	10/2014	10299	TA	*A. xylosoxidans*					
24	10/2013	10668	TA	*A. xylosoxidans*	34	10/2014	10593	TA	*A. xylosoxidans*					

*^1^TA, tracheal aspirate*.

*^2^BALF, bronchoalveolar lavage fluid*.

At least one isolate per patient was included, as well as subsequent isolates that were considered phenotypically different. The strains' names were assigned randomly to preserve the anonymity of the patients. The study protocol was approved by Ethical Comittee of the Mother and Child Health Care Institute “Dr Vukan Cupic,” (approval No. 8/6).

### Identification and phylogenetic analyses

Preliminary identification of isolates was performed by the Vitek 2 automated system (bioMérieux, Marcy l′Etoile, France) and sequencing of genes for 16S rRNA; PCR amplification of the 16S rRNA gene was performed using primers UNI 16SF (5–GAG AGT TTG ATC CTG GC-3) and UNI 16SR (5-AGG AGG TGA TCC AGC CG-3) (Jovcic et al., 2009). Species identification was performed by sequencing of *nrdA* gene as described previously (Spilker et al., 2012). The PCR products were purified by using the GeneJET PCR Purification Kit (Thermo Scientific, Lithuania) and sequenced by the MACROGEN service (Macrogen Inc., Netherlands). For 16S rRNA identification Basic Local Alignment Search Tool (BLAST, http://blast.ncbi.nlm.nih.gov/Blast.cgi) was used for searches against GenBank database. The phylogenetic inferences between *Achromobacter* spp. were obtained by MEGA version 6.0 (Tamura et al., 2013). The *nrdA* gene sequences were trimmed to 449 bp and aligned using Clustal W with default parameters. The construction of a phylogenetic tree was conducted by the maximum-likelihood (ML) method using a Tamura-Nei model. Data for the 28 reference strains were derived from pubMLST website (http://pubmlst.org/achromobacter/) and included in this analysis. Bootstrapping of 1,000 replicates was used to infer confidence levels of ML trees. The *nrdA* gene sequences from analyzed strains are deposited in GenBank BankIt2010541: KY968572–KY968640.

### Pulsed field gel electrophoresis (PFGE)

Genetic relatedness among analyzed isolates was determined by PFGE analysis of *Xba*I digested genomic DNA. Electrophoresis was carried out for 18 h using contour-clamped homogeneous electric field system (2015 Pulsafor unit; LKB Pharmacia, Sweden) in 0.5X TBE (45 mmol/l Tris base, 45 mmol/l boric acid, and 1 mmol/l EDTA pH 8), as described previously (Barton et al., 1995). A dendrogram was derived from the Ward linkage of correlation coefficients between PFGE patterns of different genotypes by using SPSS cluster analysis software (IBM Corp. Released 2012. IBM SPSS Statistics for Windows, Version 21.0. Armonk, NY: IBM Corp.). Tenover's criteria were used for the interpretation of PFGE results (Tenover et al., 1995).

### Antimicrobial susceptibility testing

Antimicrobial susceptibility testing was performed by the disk diffusion method according to recommendations of Clinical and Laboratory Standards Institute (CLSI, 2014). Isolates were tested against 10 antimicrobial agents: ceftazidime (30 μg), ceftriaxone (30 μg), aztreonam (30 μg), imipenem (10 μg), amikacin (30 μg), gentamicin (10 μg), ciprofloxacin (5 μg), levofloxacin (5 μg), tetracycline (30 μg), chloramphenicol (30 μg) (BioRad, France). For antibiotics with undefined breakpoints for *Achromobacter* spp. in current standards, breakpoints for *Pseudomonas aeruginosa* were used.

Minimal inhibitory concentrations (MICs) were determinated by microdilution testing following CLSI breakpoints for other non-*Enterobacteriaceae*. Susceptibility was tested against imipenem (4–16 μg/ml), meropenem (4–16 μg/ml), piperacillin (16–128 μg/ml), piperacillin/tazobactam (16/4–128/4 μg/ml), tetracycline (4–16 μg/ml) and trimethoprim/sulfamethoxazole (2/38–4/76 μg/ml) for all isolates, while only isolates displaying intermediate susceptibility or resistance with the disk diffusion method were tested against ceftazidime (8–32 μg/ml), ciprofloxacin (1–4 μg/ml), levofloxacin (2–8 μg/ml) and chloramphenicol (8–32 μg/ml). Experiments were done in triplicate. After 24 h incubation at 37°C, cell density was monitored by OD_600_ measurements in a microtiter Plate Reader Infinite 200 pro (MTX Lab Systems, Austria), and MIC values were determinated as the lowest concentration of antibiotic that inhibited bacterial growth.

### Detection of ESBL and MBL producing isolates

The double disc synergy test (DDST) was used to screen for extended-spectrum beta-lactamase (ESBL)-producing strains. Isolates presumed to be ESBL producers on the basis of screening test results were subjected to the phenotypic confirmatory test (Tsering et al., 2009). Only the ones positive in the confirmatory test were analyzed by molecular testing methods.

The detection of metallo-beta-lactamase (MBL) producers was carried out by disc diffusion test with imipenem (10 μg) and imipenem/EDTA (10 μg/930 μg) (Yong et al., 2002).

The PCR method was used for detection of genes encoding various beta-lactamases (ESBL, AmpC-like, MBL) in selected candidates. Sets of primers specific for the TEM, SHV, CTX-M, OXA-1, OXA-2, OXA-114, CMY-1, CMY-2, VEB-1, DHA-1, GES-7, IMP-1, IMP-2, VIM-2, NDM-1, TMB, and SIM genes used in this study are listed in Table 3.

**Table 3:** **List of primers used in this study**.

**Primers**	**Gene**	**Sequence 5′ → 3′**	**Th (°C)**	**References**
VEB-1F	*bla*_VEB−1_	CCAGATAGGAGTACAGAC	47°C	Neuwirth et al., 2006
VEB-1R		GACTCTGCAACAAATACGC		
SHVF	*bla*_SHV_	GTCAGCGAAAAACACCTTGCC	47°C	Sharma et al., 2013
SHVR		GTCTTATCGGCGATAAACCAG		
CMY-1F	*bla*_CMY−1_	GCTGCTCAAGGAGCACAGGATCCCG	61°C	Teo et al., 2012
CMY-1R		GGCACATTGACATAGGTGTGGTGCATG		
CMY-2F	*bla*_CMY−2_	ACTGGCCAGAACTGACAGGCAAA	57°C	Teo et al., 2012
CMY-2R		GTTTTCTCCTGAACGTGGCTGGC		
CTX-MF	*bla*_CTX−M_	GAAGGTCATCAAGAAGGTGCG	48°C	Sharma et al., 2013
CTX-MR		GCATTGCCACGCTTTTCATAG		
DHA-1F	*bla*_DHA−1_	GTCGCGGCGGTGGTGGAC	57°C	Teo et al., 2012
DHA-1R		CCGCACCCAGCACACCTGT		
TEMF	*bla*_TEM_	ATAAAATTCTTGAAGACGAAA	52°C	Teo et al., 2012
TEMR		GACAGTTACCAATGCTTAATCA		
GES-7F	*bla*_GES−7_	ATCTTGAGAAGCTAGAGCGCG	48°C	Teo et al., 2012
GES-7R		GTTTCCGATCAGCCACCTCT		
OXA-1F	*bla*_OXA−1_	CTTGATTGAAGGGTTGGGCG	55°C	Aubert et al., 2001
OXA-1R		AGCCGTTAAAATTAAGCCC		
OXA-2F	*bla*_OXA−2_	GAAGAAAACGCTACTCGC	47°C	Orman et al., 2002
OXA-2R		TACCCACCAACCCATAC		
OXA-114A	*bla*_OXA−114_	ACGCCTGAACCCTTTTATCC	50°C	Amoureux et al., 2013
OXA-114B		ATCGACAGGCCGCGCAGT		
IMP-1F	*bla*_IMP−1_	TGAGCAAGTTATCTGTATTC	48.5°C	Yan et al., 2001
IMP-1R		TTAGTTGCTTGGTTTTGATG		
IMP-2F	*bla*_IMP−2_	GGCAGTCGCCCTAAAACAAA	45°C	Yan et al., 2001
IMP-2R		TAGTTACTTGGCTGTGATGG		
VIM-2F4	*bla*_VIM−2_	GTTTGGTCGCATATCGCA	51°C	Ellington et al., 2007
VIM-2R4		AATGCGCAGCACCAGGAT		
NDMfullF	*bla*_NDM−1_	TCAGCGCAGCTTGTCGGCC	58°C	Teo et al., 2012
NDMfullR		ATGGAATTGCCCAATATTATG		
Trip75	*bla*_TMB−1_	ACCCGGATTGGAAGTTGAGG	54°C	El Salabi et al., 2012
Trip617R		TTCTAGCGGATTGTGGCCAC		
SIMF	*bla*_SIM_	TACAAGGGATTCGGCATC	52°C	Ellington et al., 2007
SIMR		TAATGGCCTGTTCCCATG		

### Biofilm formation

Biofilm formation assay was performed according to the method described previously (Stepanović et al., 2007), with modifications. Wells of 96-well microtiter plates (Tissue Culture Plate, Sarstedt, Germany) were filled with 180 μl Luria-Bertani broth medium and then 20 μl aliquotes of overnight cultures of *Achromobacter* spp. strains (adjusted to the 0.5 McFarland standard) were added. All strains were tested in triplicate. Sterile medium tested in triplicate was used as negative control. Microtiter plates were incubated aerobically for 48 h at 37°C. After incubation and washing (three times with phosphate-buffered saline, PBS; pH 7.2) remaining bacteria were fixed by drying at 65°C for 30 min. For staining and visualization of biofilm, 0.1% crystal violet (HiMedia Labs Pvt. Ltd., India) was used (30 min at room temperature). The stain was rinsed by washing three times with 1X PBS and then resolubilised with 96% ethanol and acetone (4:1). Quantification of biofilm formation was done by measuring absorbance at 595 nm using Plate Reader Infinite 200 pro (MTX Lab Systems, Austria).

On the basis of biofilm formation, *Achromobacter* spp. isolates were divided into four classes: no biofilm producer (N), weak (W), moderate (M), and strong (S) biofilm producer (Stepanović et al., 2007).

### Motility assay

Swimming assay of *Achromobacter* strains was performed on plates containing tryptone (10 g/l), NaCl (5 g/l), and 0.3% (wt/vol) agarose (Trancassini et al., 2014). Plates were inoculated with one colony of each strain and incubated at 37°C for 18 h. For defining the motility of a specific strain, the diameter of the resulting concentric ring, expressed in millimeters, was measured. If growth diameter could not be measured isolates were classified as non-motile.

### Mucin adhesion ability

The binding of bacterial cells to mucin was tested according to the method described previously (Muñoz-Provencio et al., 2009), with modifications. The wells of microtiter plates (Tissue Culture Plate, Sarstedt, Germany) were coated at 4°C for 24 h with 200 μl of porcine stomach mucin type II (Sigma, Germany). The main component of porcine stomach mucin is MUC2, which is homologous to human MUC5AC mucin produced by superficial epithelium in trachea and bronchi (Turner et al., 2007; Kreda et al., 2012). Mucin was resuspended in 50 mM carbonate buffer (pH 9.6; 30 mg/ml) and the same volume of carbonate buffer was added to the control wells (without mucin). All wells were washed three times with 1X PBS. Afterwards, PBS with 1% Tween 20 was added to saturate the uncoated binding places. After 1 h incubation at room temperature, the wells were washed once more with 1X PBS and, subsequently, 200 μl of each bacterial suspension in 1X PBS was added (the suspension was adjusted to 0.5 McFarland standard). Bacterial suspensions were prepared by resuspension of overnight cultures in PBS. Each strain was tested in triplicate in both coated and control wells. After incubation for 2 h at 37°C, the wells were washed three times with 1X PBS containing 0.05% Tween 20 to remove nonadherent cells. Fixation of mucin-bound bacterial cells was performed by drying the plate at 65°C for 45 min. Subsequently, 200 μl aliquots of crystal violet (HiMedia Labs Pvt. Ltd., India) were added to the wells in a final concentration of 0.1 mg/ml. After 45 min, the solution was decanted and the unbound stain was removed by three washes with 1X PBS, and finally citrate buffer (50 mM; pH 4) was added to dissolve the stain bound to the bacterial cells. After 1 h incubation at room temperature, the absorbance was measured at 595 nm using the Labsystems Multiskan RC plate reader (MTX Lab Systems, Austria).

### Collagen-and fibronectin-binding assay

The wells of Maxisorb plates (Nunc, Roskilde, Denmark) were coated with type I collagen (from rat tail, BD Bioscience, New Jersey, United States) (100 μg/ml) or human fibronectin (Serva, Heidelberg, Germany) (100 μg/ml) for 16 h at 4°C. The collagen-binding ability of the selected strains was tested according to Miljkovic et al. (2015), while their ability to bind fibronectin was assayed as previously described by Ahmed et al. (2001). After immobilization, the wells were washed with PBS and blocked with 2% BSA in PBS. When the BSA solution was removed, the wells were washed with PBS and the test cultures (100 μl, 10^8^ CFU/ml) were added. Plates were incubated on an orbital platform shaker for 3 h at 37°C. Then, nonadherent cells were removed by washing the wells three times with 200 μl of PBS. The adherent cells were fixed at 60°C for 20 min and stained with crystal violet (100 μl/well, 0.1% solution) for 45 min. Wells were subsequently washed three times with PBS to remove the excess stain. The stain bound to the cells was dissolved in 100 μl of citrate buffer (pH 4.3) and the absorbance was measured at 595 nm after 45 min using the microtiter plate reader. Results were expressed as the mean of six replicates relative to those of the same non-coated wells. Laboratory strain *Lactococcus lactis* BGKP1 was used as a positive control.

### Elastase assay

Supernatants of overnight cultures were mixed with phosphate buffer (0.1 M, pH 6.3) in a 2:1 ratio. Elastin-Congo red (Sigma, St. Louis, United States) was added to the final concentration of 2 mg/ml and the mixtures were incubated at 37°C with shaking (200 rpm) for 1 week and subsequently centrifuged at 10,000 × g for 10 min. The absorbance was measured at 495 nm and the values were subtracted from a blank containing NaCl (0.9%) incubated without supernatant (Jakobsen et al., 2013). *Pseudomonas aeruginosa* PAO1 was used as a positive control.

### Summarising the virulence properties data

In order to summarize virulence potential data for the analyzed strains heat map was constructed using Rx64 3.2.3. software.

A dendrogram was created by clustering algorithm that related rows by similarity. The results were approximated on the relative scale ranging from 0 (blue) as the lowest values, progressing to white, then to 100 (red) as the highest values. Analyzed strains were separated into three clusters which summarize each virulence factor potential.

### Statistical analysis

For comparing differences in biofilm formation, motility, mucin-, collagen-, and fibronectin-adhesion ability between CF and non-CF isolates, Mann-Whitney test was used. Correlations between tested parameters were performed using Spearman's test. Results are represented as box-plots. To compare the proportion of motile strains between CF and non-CF patients, Pearson's chi-squared test was used. All statistical analyses and drawing of graphs and dendrograms for PFGE analysis were performed in SPSS 21.0 for Windows.

## Results

### *Achromobacter* spp. isolates and *nrdA* gene sequence clustering

**Figure 1:**
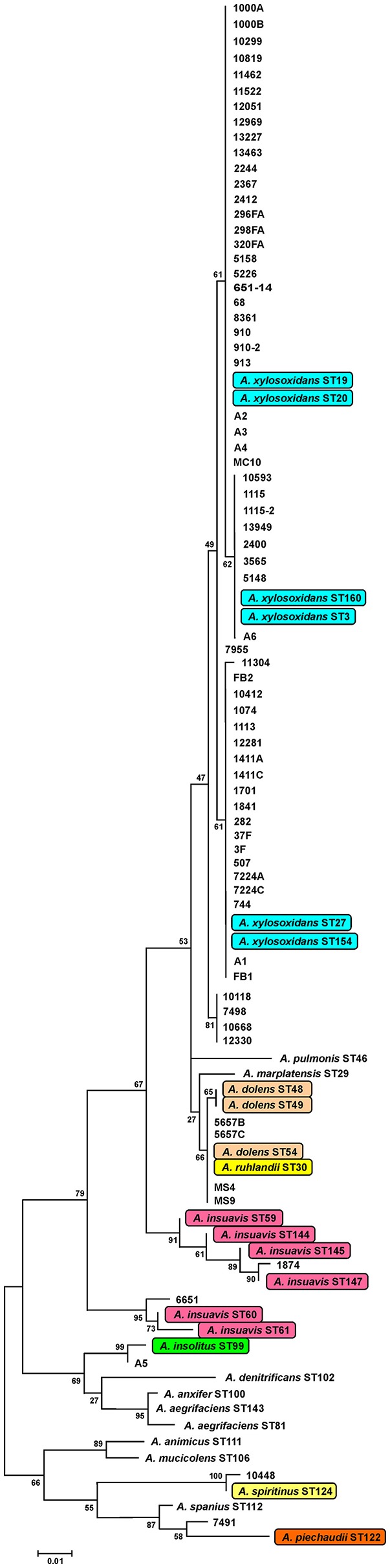
**Maximum-likelihood phylogenetic tree based on definite article the ***nrdA*** gene sequences (449 bp alignment) of ***Achromobacter*** type strains. Data for reference strains were derived from the pubMLST website (http://pubmlst.org/achromobacter/)**. Type strains of species represented amnong strains from the study are highlighted.

Identification by the Vitek 2 system and 16S rRNA genes sequencing revealed that all 69 isolated strains belonged to *Achromobacter* spp. Species-level identification performed by the *nrdA* gene sequencing showed that *A. xylosoxidans* was the most prevalent species (60/69, 87.0%). The *nrdA* gene sequence clustering successfully segregated 60 isolates of *A. xylosoxidans* into 4 defined clusters (Figure 1). *Achromobacter* species other than *A. xylosoxidans* were identified as *A. dolens/ A. ruhlandii* (5657B, 5657C, MS4, and MS9 CF isolates, 4/69, 5.8%, these species were undistinguishable with used the *nrdA* gene sequences), *A. insuavis* (1874 CF and 6651 non-CF isolate, 2/69, 2.9%), *A. spiritinus* (10448 CF isolate, 1/69, 1.4%), *A. insolitus* (A5 non-CF isolate, 1/69, 1.4%) and *A. piechaudii* (7491 non-CF isolate, 1/69, 1.4%) (Figure 1).

### Genotype analysis by PFGE

**Figure 2:**
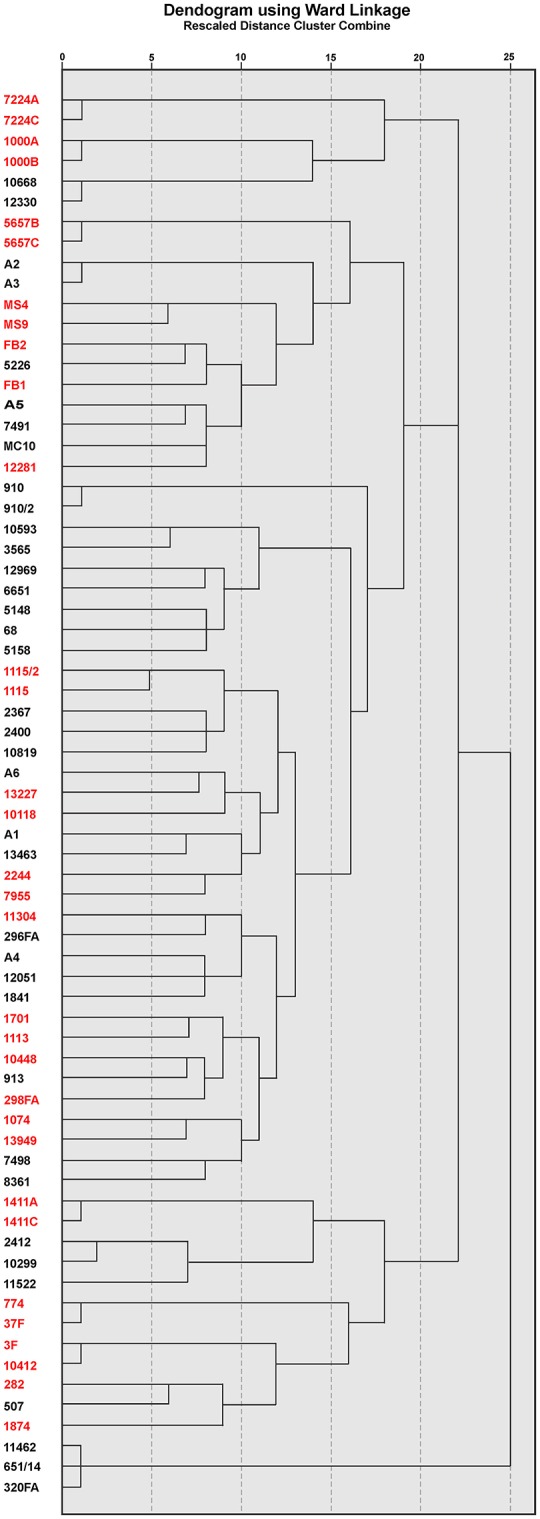
**Dendrogram created according to PFGE profile of ***Xba***I digested genomic DNA of CF (red) and non-CF (black) ***Achromobacter*** spp. isolates**.

According to the obtained PFGE *Xba*I fingerprint, a dendrogram was constructed for all isolates and shown in Figure 2. The PFGE analysis revealed considerable genetic heterogeneity between strains within both CF and non-CF group, as well as between groups. The CF isolates were genetically more heterogeneous comparing to the non-CF group. However, three clusters of epidemiologically related isolates were detected. Possible nosocomial transmission was detected between two CF patients carrying *A. xylosoxidans* strains 3F and 10412 with the same genotype according to PFGE (Figure 2); both patients were treated in the CF unit a couple of months apart. Secondly, three non-CF patients treated at the medical ICU were colonized with *A. xylosoxidans* strains 11462, 651/14, and 320FA sharing identical banding patterns (Figure 2). Finally, *A. xylosoxidans* strains A2 and A3, isolated from two non-CF patients treated at the medical ICU, were indistinguishable as shown by PFGE (Figure 2).

### Antimicrobial susceptibility testing

**Figure 3:**
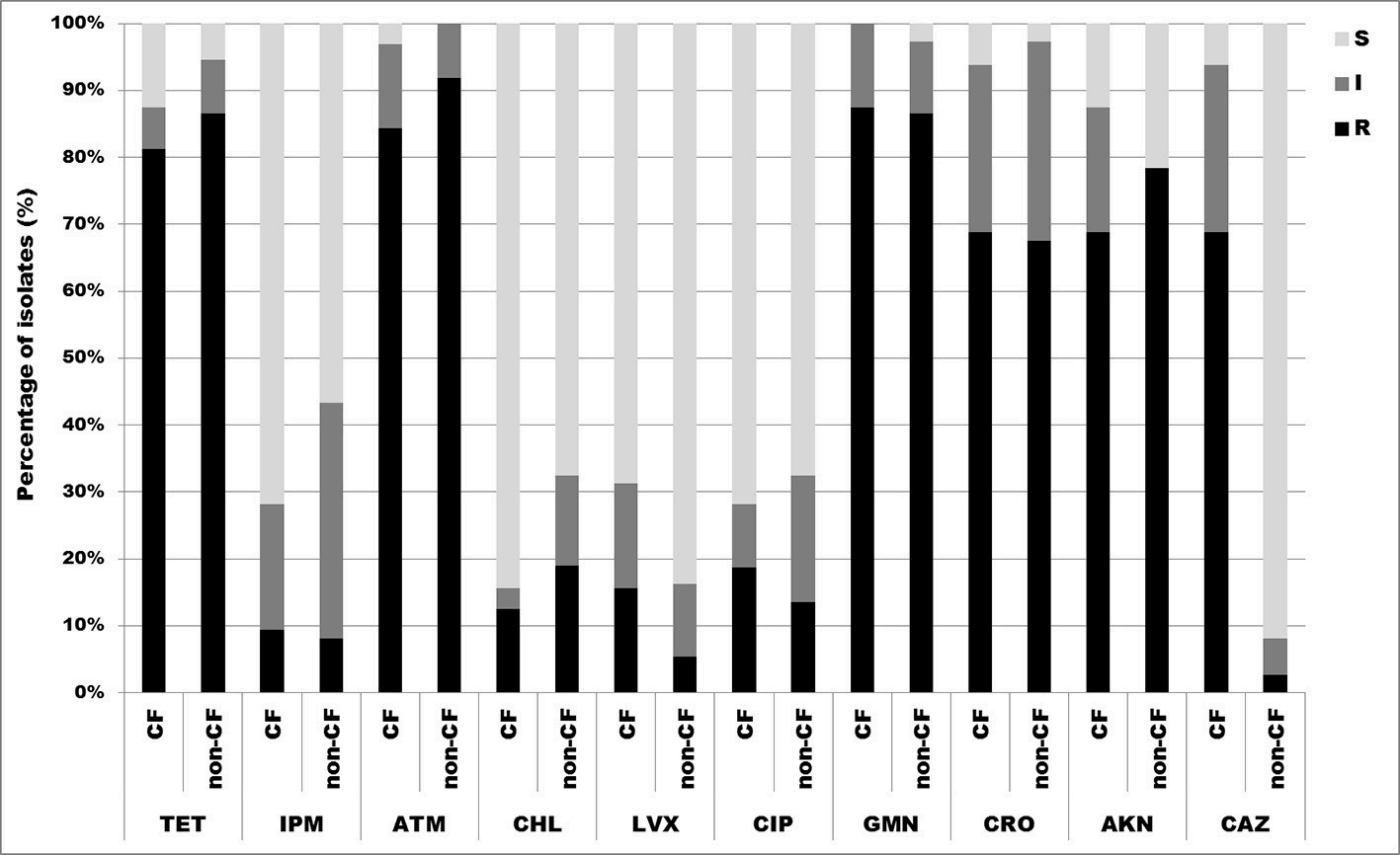
**Distribution of antibiotic resistances among CF and non-CF ***Achromobacter*** spp. isolates determined by disk diffusion method. TET, tetracycline; IPM, imipenem; ATM, aztreonam; CHL, chloramphenicol; LVX, levofloxacin; CIP, ciprofloxacin; GMN, gentamicin; CRO, ceftriaxone; AKN, amikacin; CAZ, ceftazidime; S, sensitive; I, intermediate; R, resistant**.

All 69 isolates were tested for susceptibility to 10 antimicrobial agents by the disk diffusion method. The results showed high overall frequency of resistance to aztreonam 88.4% (27/32, 84.4% CF isolates and 34/37, 91.9% non-CF isolates), gentamicin 87.0% (28/32, 87.5% CF isolates and 32/37, 86.5% non-CF isolates), tetracycline 84.1% (26/32, 81.3% CF isolates and 32/37, 86.5% non-CF isolates), amikacin 73.9% (22/32, 68.8% CF isolates and 29/37, 78.4% non-CF isolates) and ceftriaxone 68.1% (22/32, 68.8% CF isolates and 25/37, 67.6% non-CF isolates). Overall resistance to imipenem, chloramphenicol, levofloxacin, ciprofloxacin and ceftazidime was not significant (less than 20%) (Figure 3). *A. dolens*/*A. ruhlandii* isolates showed multiresistant patterns according to the disc diffusion method. Strains MS4 and MS9 were resistant to most of the tested antibiotics except levofloxacin, ciprofloxacin and ceftazidime (MS4), and levofloxacin, ciprofloxacin, imipenem, and chloramphenicol (MS9), while 5657B and 5657C isolates were resistant to tetracycline, aztreonam, gentamicin, amikacin, and ceftriaxone. *A. insuavis* isolates were resistant to imipenem and amikacin (CF isolate marked as 1874) and aztreonam, gentamicin, ceftriaxone (non-CF isolate marked as 6651). *A. spiritinus* isolate was resistant only to gentamicin, *A. insolitus* to aztreonam, while *A. piechaudii* isolate was sensitive to all tested antibiotics.

**Figure 4:**
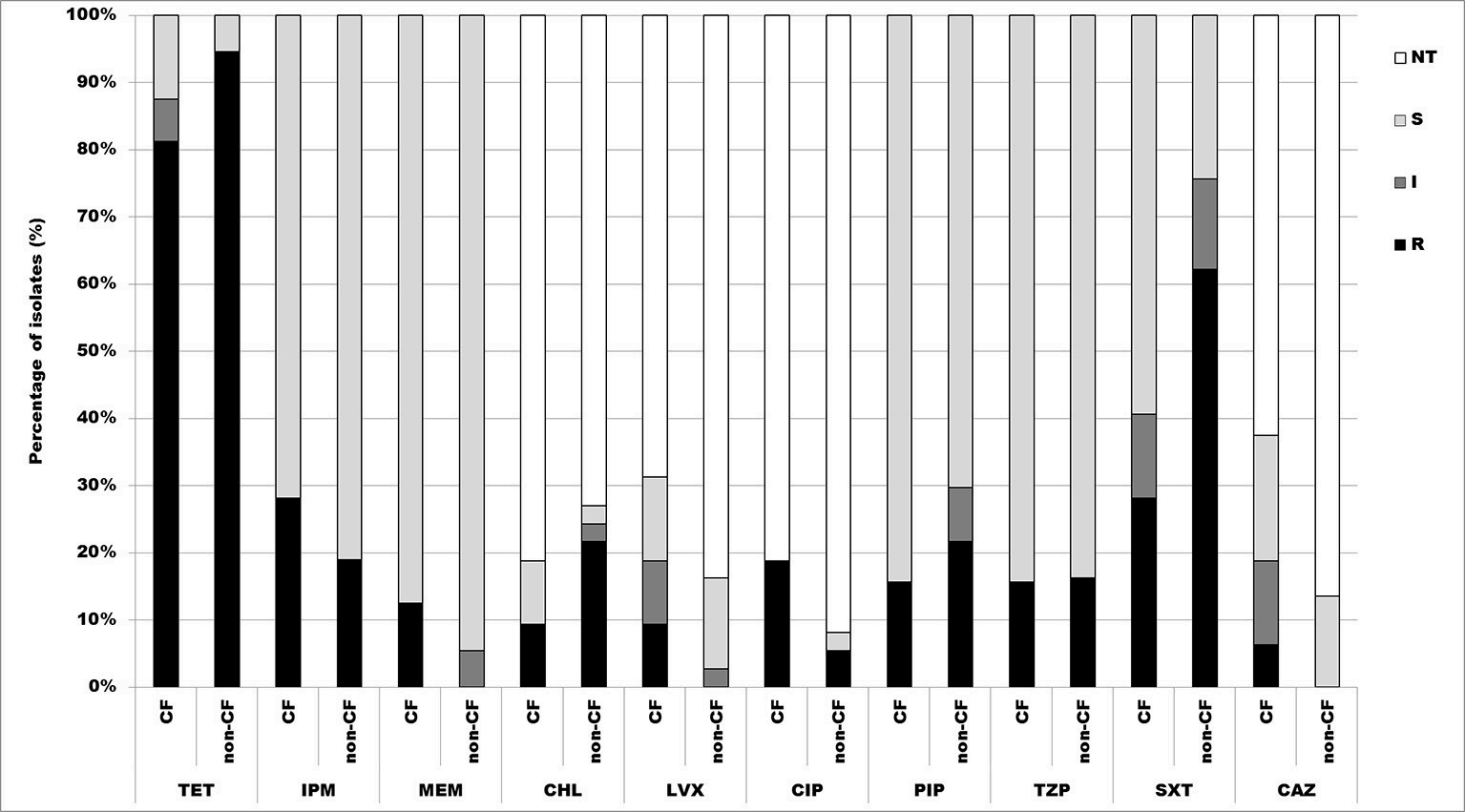
**Distribution of antibiotic resistances among CF and non-CF ***Achromobacter*** spp. isolates determined by microdilution method. TET, tetracycline; IPM, imipenem; MEM, meropenem; CHL, chloramphenicol; LVX, levofloxacin; CIP, ciprofloxacin; PIP, piperacillin; TZP, piperacillin/tazobactam; SXT, trimethoprim/sulfamethoxazole; CAZ, ceftazidime; NT, not tested; S, sensitive; I, intermediate; R, resistant**.

According to the results obtained by microdilution test, 88.4% (61/69) of isolates were resistant to tetracycline, thereof 81.3% (26/32) CF isolates, and 94.6% (35/37) non-CF isolates. There were 46.4% (32/69) of isolates resistant to trimethoprim/sulfamethoxazole, 28.1% (9/32) CF isolates, and 62.2% (23/37) non-CF isolates. 23.2% (16/69) were resistant to imipenem, 28.1% (9/32) CF isolates and 18.9% (7/37) non-CF isolates. Percentage of isolates resistant to piperacillin was 18.8% (13/69), 15.6% (5/32) CF isolates, and 21.6% (8/37) non-CF isolates, while 15.9% (11/69) were resistant to piperacillin/tazobactam, 15.6% (5/32) CF isolates and 16.2% (6/37) non-CF isolates. 5.8% (4/69) were resistant to meropenem, all of them belonging to the CF group. Percentages of resistant CF and non-CF isolates are presented in Figure 4.

Determination of MIC values for antibiotics used in disk diffusion method was performed only for resistant and intermediate strains. Obtained results revealed that 15.9% (11/69) isolates were resistant to chloramphenicol, 9.4% (3/32) CF isolates and 21.6% non-CF isolates. 11.6% (8/69) were resistant to ciprofloxacin, 18.7% (6/32) CF isolates and 5.4% (2/37) non-CF isolates. Only 4.3% (3/69) were resistant to levofloxacin, 9.4% (3/32) CF isolates; and 2.9% (2/69) isolates were resistant to ceftazidime 6.2% (2/32) of CF isolates (Figure 4).

*A. dolens*/*A. ruhlandii* isolates showed multiresistant phenotype according to the microdilution test, except 5657B isolate which was resistant only to tetracycline and trimethoprim/sulfamethoxazole. *A. insuavis* 1874 isolate was resistant to imipenem and trimethoprim/sulfamethoxazole. *A. spiritinus, A. piechaudii*, and non-CF isolate *A. insuavis* 6651 were sensitive to all tested antibiotics.

### ESBL and MBL activity

DDST assay and phenotypic confirmatory test revealed that 56.3% (18/32) CF and 48.6% (18/37) non-CF isolates were putatively ESBL positive. Imipenem and imipenem/EDTA test revealed presence of 28.1% (9/32) CF and 43.2% (16/37) non-CF isolates as putative MBL-producers. *A. insolitus* and *A. dolens*/*A. ruhlandii* (5657B, 5657C, and MS9) isolates were phenotypically MBL-positive. 12.5% (4/32) CF and 16.2% (6/37) non-CF isolates were both ESBL and MBL positive. *A. dolens*/*A. ruhlandii* MS4, *A. insuavis, A. spiritinus* and *A. piechaudii* were detected as neither ESBL nor MBL-producers. Isolates selected by phenotypic tests were analyzed for the presence of common ESBL or MBL genetic determinants. Two non-CF isolates (A3 and A4) were confirmed for the presence of *bla*_CMY−2_ gene which encodes AmpC beta-lactamase, a clinically important cephalosporinase.

### Biofilm formation

Under the tested conditions, only two CF isolates (*A. spiritinus* 10448 and *A. dolens*/*A. ruhlandii* MS9) out of 69 tested (2/69, 2.9%) were unable to produce biofilm (N class). 23.2% (16/69) isolates were classified as weak (W) biofilm producers, thereof 18.8% (6/32) from CF and 27.0% (10/37) from non-CF group. 44.9% (31/69) isolates were moderate (M) biofilm producers; of those, 46.9% (15/32) were CF isolates, and 43.2% (16/37) non-CF isolates. 29.0% (20/69) isolates were strong (S) biofilm producers, 28.1% (9/32) of them were CF isolates, and 29.7% (11/37) non-CF.

*A. insuavis, A. piechaudii*, and *A. dolens*/*A. ruhlandii* 5657B were classified as weak (W) biofilm producers. *A. insolitus* and *A. dolens*/*A. ruhlandii* 5657C were moderate (M) biofilm producers, while *A. dolens*/*A. ruhlandii* MS4 was a strong (S) biofilm producer.

No statistical differences were observed in biofilm formation between the CF and non-CF group of isolates.

### Motility assay

**Figure 5:**
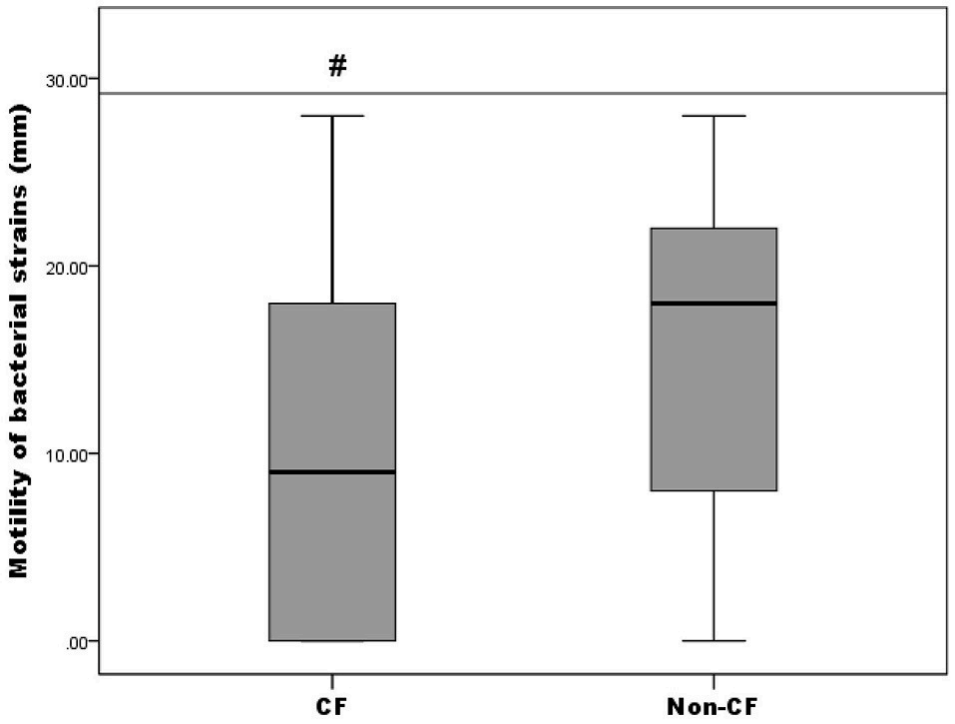
**Motility of CF isolates relative to non-CF isolates; # denotes statistical significance relative to non-CF isolates**.

Motility of the isolates was tested by a swimming motility assay and the results were expressed as the diameter of growth ring in millimeters (data not shown). It was established that 82.61% (57/69) were positive for swimming motility, thereof 71.87% (23/32) from the CF group and 91.89% (34/37) from the non-CF group. Only *A. insuavis* 6651 was positive for swimming motility among the non-*xylosoxidans Achromobacter* isolates. To compare the proportion of motile strains between CF and non-CF patients, the Pearson's chi-square test was used [*p*(χ^2^) = 0.029]. Different levels of motility between bacteria isolated from CF and non-CF patients are presented in Figure 5. Strains isolated from the non-CF patients showed a higher degree of motility relative to the CF strains, according to the Mann-Whitney test (*p* = 0.044) (Figure 5).

### Mucin-, collagen-, and fibronectin-adhesion ability and elastase activity

**Figure 6:**
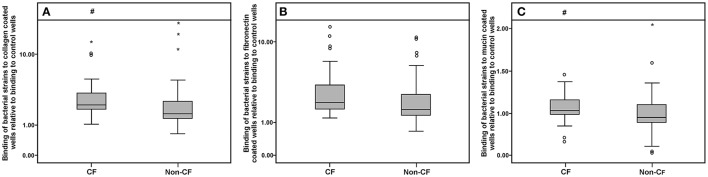
Collagen (A), fibronectin (B), and mucin (C) binding abilities of CF and non-CF isolates; ◦ and ^*^ represent extreme and outlier values, respectively; # denotes statistical significance relative to non-CF isolates.

Collagen-, fibronectin-, and mucin-binding ability (expressed relatively to control non-coated wells in the microtiter plate) of bacterial isolates are shown in Figure 6. Strains derived from the CF patients exhibited higher binding affinity to collagen and mucin compared to the non-CF strains (*p* < 0.05, according to the Mann-Whitney test), while for fibronectin binding, only a statistical trend (*p* < 0.1) was observed (Figure 6). Among the non-*xylosoxidans Achromobacter* species, *A. spiritinus* showed the highest binding affinity to collagen, while *A. insuavis* 1874 had the highest binding affinity to fibronectin. None of the isolates showed elastase activity.

### Comparative evaluation of *Achromobacter* spp. virulence

**Figure 7:**
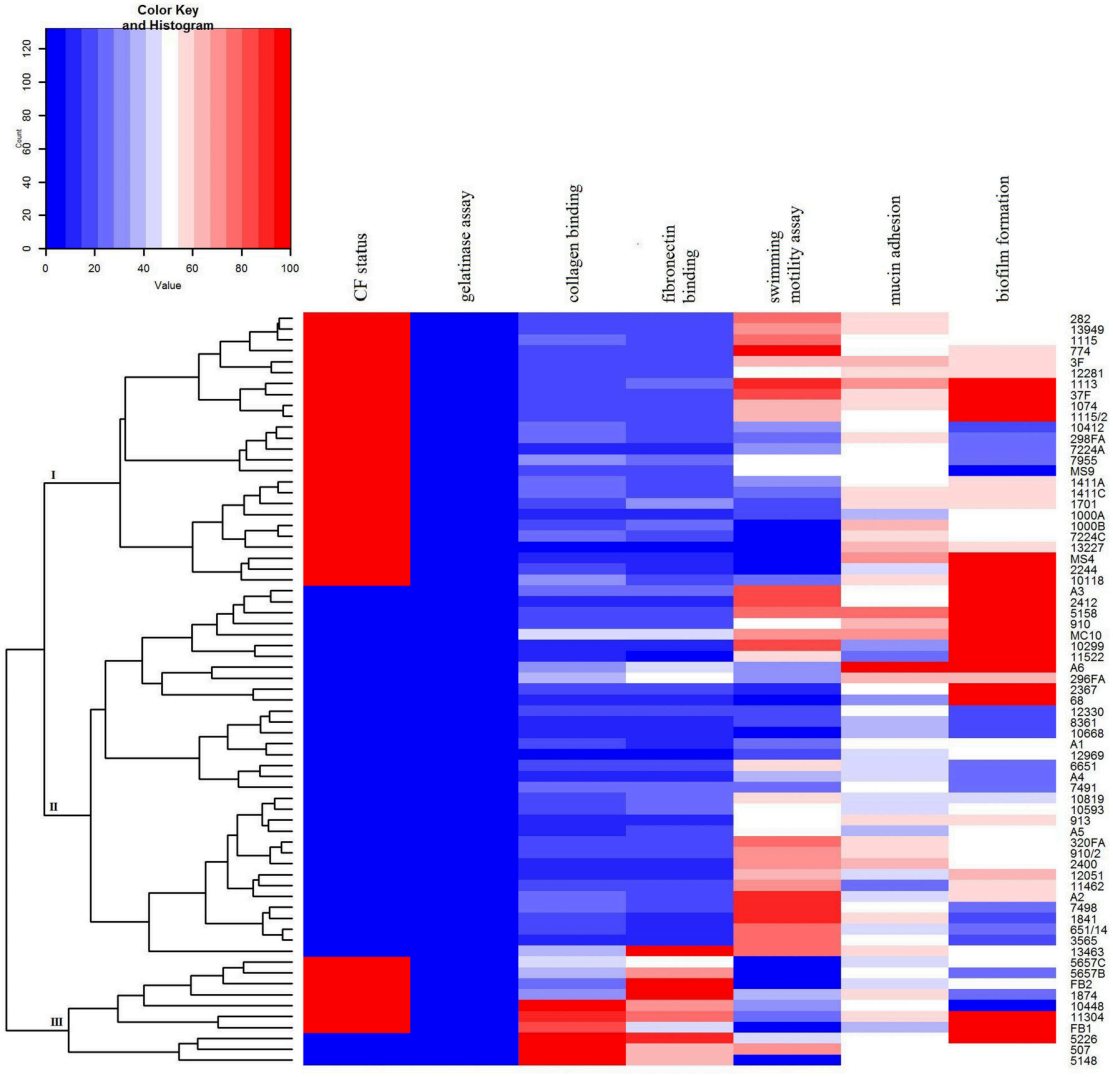
**Heat map demonstrating the distribution of virulence traits in CF- and non-CF ***Achromobacter*** isolates. Results were approximated on the relative scale ranging from 0 (blue) as the lowest values, progressing to white, then to 100 (red) as the highest values**.

A heat map demonstrating distribution of the virulence characteristics analyzed in this study was constructed in order to summarize the overall virulence potential of CF and non-CF isolates (Figure 7). According to the heat map, all tested isolates can be divided into three distinct clusters: CF group (cluster I), non-CF group (cluster II), and the cluster III comprising the strains considered to be the most virulent, including 7 CF and 3 non-CF isolates (Figure 7).

## Discussion

Epidemiology, antibiotic resistance and virulence traits of 69 *Achromobacter* spp. isolates recovered from clinical samples were analyzed in this study. *A. xylosoxidans* was the most prevalent species among the strains of both CF (81.3%) and non-CF origin (91.9%). This finding is in correlation with data reported in other studies (Ridderberg et al., 2012; Perez-Losada et al., 2013; Spilker et al., 2013). The CF patients followed at the National CF Center of Serbia were also colonized with *A. dolens/A. ruhlandii, A. insuavis*, and *A. spiritinus* strains, while the non-CF group of isolates was somewhat less heterogeneous with *A. insuavis, A. insolitus*, and *A. piechaudii* strains detected beside *A. xylosoxidans. A. ruhlandii* has been reported as the second most common species in CF patients, with a very high prevalence rate (23.5%) in the USA and only 3% in the UK (Spilker et al., 2013; Coward et al., 2015). Nevertheless, the epidemiology and possible clinical significance of non-*xylosoxidans Achromobacter* species in CF and non-CF patients are generally unknown.

The genotypic analysis by PFGE revealed considerable genetic heterogeneity, indicating that most of the patients harbored their own unique strains. A marked diversity of *Achromobacter* spp. genotypes was also detected in other CF centers (Pereira et al., 2011; Amoureux et al., 2013), suggesting that the majority of the isolates probably originate from environmental sources.

However, in our study, three *A. xylosoxidans* strains were involved in instances of possible clonal distribution; one was shared between two CF patients treated in the CF unit a couple months apart, and the other two were isolated from non-CF patients treated at the medical ICU. Although rare, cross-transmission of *A. xylosoxidans* has been detected in some CF centers (Van Daele et al., 2005; Pereira et al., 2011). There are limited data available on the cross-transmission capacity of non-*xylosoxidans Achromobacter* strains, except those concerning the spread of a particular clone of *A. ruhlandii* among CF patients in Denmark (Hansen et al., 2013).

Colonization of the CF lung by multiresistant *Achromobacter* strains, primarily *A. xylosoxidans*, has proved to be a treatment challenge. The data on resistance profiles and underlying resistance mechanisms of other *Achromobacter* spp. are very limited. Resistance phenotypes for aztreonam, gentamicin and amikacin were similar to data obtained in an Italian CF center (Trancassini et al., 2014), and weren't surprising due to *A. xylosoxidans* intrinsic resistance to aminoglycosides and aztreonam (Rolston and Messer, 1990; Almuzara et al., 2010).

Although *A. xylosoxidans* bears innate resistance to aminoglycosides, literature data suggest that some *Achromobacter* spp. strains are susceptible to these drugs (Bador et al., 2016). This is in accordance with our study and could explain detected susceptibility to aminoglycosides among studied isolates.

Since disk diffusion method is not validated by the CLSI nor EUCAST for most of the nonfermenting Gram-negative bacilli antibiotic susceptibility was additionaly tested by microdilution method. Results obtained by the disk diffusion correlated to those from microdilution method for some of the tested antibiotics (chloramphenicol, levofloxacin, and ceftazidime). About one half of tested isolates were resistant to trimethoprim/sulfamethoxazole, which is often used for treating infections caused by *A. xylosoxidans*. Piperacillin, piperacillin/tazobactam, and carbapenems displayed the best antibacterial effect against the tested strains, as was the case in some of the other studies (Almuzara et al., 2010). Meropenem had higher efficiency in comparison to imipenem, confirming its status of most effective carbapenem (Almuzara et al., 2010). Among non-*xylosoxidans Achromobacter* strains, *A. dolens*/*A. ruhlandii* showed a multiresistant phenotype. Low specifity and positive predictive value of conventional phenotypic method for detecting MBL producers revealed in our study had previously been described for other Gram-negative non-fermenting bacilli (Samuelsen et al., 2008).

Despite the increasing importance of *Achromobacter* spp. little is known about the virulence factors associated with these bacteria. So, this study aimed to explore and analyze the virulence armament of *Achromobacter* clinical strains through expression of characteristics such as biofilm formation, motility, production of elastase and adhesion capacity to collagen, fibronectin and mucin. These bacterial features are generally considered to be important for *in vivo* colonization and infection (Brogden et al., 2007). The ability to form biofilms contributes significantly to increased bacterial resistance to antimicrobials and antiseptics, resulting in treatment failure and persistance of infection. Among the tested isolates, about one third were classified as strong biofilm producers, including *A. dolens*/*A. ruhlandii* MS4 strain that was the only non-*xylosoxidans Achromobacter* species from the study in this group. The ability of *Achromobacter* spp. to form biofilms was demonstrated earlier (Jakobsen et al., 2013), and in some CF centers as many as half of the tested strains were strong biofilm producers (Trancassini et al., 2014).

Given the described correlation between the loss of swimming motility and pathogenesis of CF lung infections, we assessed the presence of this trait in our isolate collection. CF isolates from this study were less motile, in tested conditions, compared to non-CF isolates. We could assume that diminished motility among our CF isolates might serve as an competitive advantage in establishing an infection in the airways, as was shown for some other Gram-negative non-fermenting bacilli of CF origin (Wolfgang et al., 2004). No correlation was found between swimming phenotype and biofilm formation, which has also been shown in the study from an Italian CF center (Trancassini et al., 2014).

Mucus hyperproduction in the airways is one of the hallmarks of the chronic airway diseases, including CF. To the best of our knowledge, this study is the first to examine the mucin binding ability of *Achromobacter* spp. Higher afinity of CF isolates to mucin compared to non-CF isolates was not surprising and might be the result of adaptive responses to mucus accumulation in the lungs of CF patients. Additionally, binding ability of CF isolates to collagen was significantly higher comparing to non-CF isolates, while for fibronectin binding only a statistical trend (*p* < 0.1) was observed. Higher affinity of CF isolates was expected as in CF lungs deposition of collagen and fibronectin is increased (Wilson and Wynn, 2009).

Unraveling of virulence traits indicated that ten isolates stood out from the rest in terms of analzyed phenotypes (cluster III, Figure 7), including *A. xylosoxidans, A. dolens*/*A. ruhlandii, A. spiritinus*, and *A. insuavis* strains. In addition, *A. dolens*/*A. ruhlandii* strains showed multiresistant patterns. The isolates belonging to the cluster III had a higher binding affinity for collagen and fibronectin as well as strong biofilm formation properties, while motility of most strains in this group was very low or they were non-motile, in general.

In summary, this study analyzed epidemiology, drug resistance and virulence potential of *Achromobacter* species collected from CF and non-CF patients receiving care in a large tertiary care pediatric hospital in Serbia. *A. xylosoxidans* was the most common species retrieved, but several non-*xylosoxidans Achromobacter* spp. were also included in the study, which might enhance the rather limited exhisting data on various features of this species. The isolates of CF origin were less motile, but exhibited higher binding affinity to mucin, collagen and trend for higher binding affinity to fibronectin.

## Author contributions

Conceived and designed the experiments: BJ, BF, and MK. Performed the experiments: MM, BF, KN, and MK. Analyzed the data: BF, BJ, JL, and MM. Acquisition of the clinical isolates: ZV. All of the authors were involved in drafting and/or revising of the manuscript; approving the final version of the manuscript and agreeded to be accountable for all aspects of the work in ensuring that questions related to the accuracy or integrity of any part of the work are appropriately investigated and resolved.

### Conflict of interest statement

The authors declare that the research was conducted in the absence of any commercial or financial relationships that could be construed as a potential conflict of interest.
